# Short-term reduction in e-cigarette dependence using a mobile health app

**DOI:** 10.18332/tpc/225571

**Published:** 2026-10-15

**Authors:** Shada Alodaini, Rana Altabee, Ghadi Alrabai, Remaz M. Alrabie, Lina Alshehri, Raghad N. Alsolami

**Affiliations:** 1 Department of Respiratory Therapy King Saud bin Abdulaziz University for Health Sciences, Ministry of National Guard Health Affairs Jeddah Saudi Arabia; 2 King Abdullah International Medical Research Center Jeddah Saudi Arabia; 3 Ministry of the National Guard-Health Affairs Jeddah Saudi Arabia

**Keywords:** electronic cigarettes, vaping cessation, mobile health, nicotine dependence, Saudi Arabia

## Abstract

**Introduction:**

E-cigarette use has increased rapidly among young adults in Saudi Arabia, yet culturally adapted cessation tools remain scarce. Mobile health (mHealth) interventions may offer scalable support, but evidence for vaping-specific digital tools, particularly in Middle Eastern populations, remains limited. We aimed to assess within-person changes in e-cigarette dependence over a 30 day period of using a mobile cessation application among young adult exclusive e-cigarette users in Saudi Arabia, using the Penn State Electronic Cigarette Dependence Index (PS-ECDI) and its item-level behavioral indicator.

**Methods:**

A prospective, single-arm, pre-post study was conducted in Jeddah, Saudi Arabia, between 2023 and 2024. Young adults aged 18–40 years who were exclusive e-cigarette users, owned a smartphone, and could read English were recruited through online platforms. Of 272 individuals screened, 123 were enrolled, and 88 participants completed both pre- and post-intervention assessments and were included in the primary analysis. Participants used the Stay Quit Coach (SQC) mobile application for 30 days. The app includes behavioral guidance, craving-management tools, tracking features, and motivational messaging. The primary outcome was change in nicotine dependence as measured by the PS-ECDI. Secondary outcomes included item-level dependence indicators (cravings, urge intensity, use frequency) and behavioral markers of cessation readiness. Repeated-measures ANOVA and paired t-tests evaluated within-person change.

**Results:**

Participants were predominantly aged 18–25 years (63.9%) and male (63.3%). Following 30 days of app use, significant reductions were observed across nearly all dependence indicators. Mean PS-ECDI scores decreased by 43%, accompanied by marked declines in daily use frequency, cravings, withdrawal symptoms, and difficulty refraining in restricted places. Repeated-measures ANOVA demonstrated a significant effect of time on dependence scores (p<0.001). Improvements were consistent across participants, with only minimal worsening observed in a minority.

**Conclusions:**

Use of a structured mobile cessation app was associated with substantial reductions in vaping dependence among young adults in Saudi Arabia. These findings provide early evidence supporting mHealth interventions as accessible, scalable tools for vaping cessation in regions where tailored cessation services are limited. Randomized trials with longer follow-up are needed to evaluate durability and long-term abstinence.

## Introduction

Tobacco use remains one of the leading preventable causes of morbidity and mortality worldwide, accounting for more than 8 million deaths annually, according to the World Health Organization (WHO)^1^. The rapid rise of electronic cigarettes (e-cigarettes) has introduced new public health challenges. These devices deliver nicotine through aerosolized vapor, typically containing propylene glycol, glycerin, flavorings, and other additives^2^. Although often marketed as safer alternatives to combustible cigarettes, growing evidence shows that e-cigarettes pose significant health risks, particularly for young adults. Nicotine exposure during adolescence and early adulthood is associated with impaired learning, reduced attention, increased impulsivity, and greater vulnerability to addiction, in addition to respiratory disease, oral pathology, cardiovascular dysfunction, and potential neurocognitive effects^3,4^. E-cigarette use has expanded rapidly among youth and young adults globally, including in Saudi Arabia. Recent regional estimates indicate that roughly one-quarter of adults in Saudi Arabia have tried e-cigarettes, with young adults representing the most common users^5,6^. Social influences, flavor availability, perceived stress relief, and misconceptions about reduced harm contribute to this trend. Many young adults also believe that e-cigarettes aid smoking cessation, despite evidence that nicotine concentrations in electronic nicotine delivery systems (ENDS) can equal or exceed those in combustible cigarettes^7^. These misperceptions highlight the need for evidence-based cessation tools tailored to this demographic.

Although e-cigarette use is rising, a substantial proportion of users express a desire to quit. US national data suggest that nearly two-thirds of adult vapers intend to stop vaping^8^, and similar motivations have been reported among young adults in Saudi Arabia^9^. Despite this demand, effective cessation interventions specifically targeting e-cigarette use remain limited. Most existing programs are designed for traditional tobacco cessation and do not account for the distinct behavioral, social, and psychological patterns associated with vaping^10^. This gap is particularly evident in Saudi Arabia, where culturally adapted and Arabic-language cessation support is scarce. Digital health interventions have emerged as promising strategies for substance cessation^11^. With smartphone ownership now nearly universal due to the country’s digital transformation^12^, mobile health (mHealth) applications provide an accessible, scalable, and cost-effective platform for delivering behavioral interventions. Across health domains, including physical activity, diabetes management, stress reduction, and alcohol use, mobile applications have demonstrated potential to modify behavior, offer real-time feedback, enhance motivation, and support self-monitoring^13^. These features are particularly appealing to young adults, who are highly engaged with mobile technologies.

Evidence for mobile-based nicotine cessation continues to grow. Several randomized trials have shown that smoking-cessation apps can reduce cigarette consumption and improve abstinence rates compared with standard care, with adherence emerging as a key predictor of success^14^. However, the broader digital cessation literature is mixed. For example, a recent meta-analysis by Guo et al.^15^ found that smartphone apps alone did not significantly improve abstinence rates, citing substantial heterogeneity in design quality and user engagement. Similar concerns apply to vaping-specific tools: many commercially available apps do not adhere to established cessation guidelines or incorporate validated behavioral strategies, raising questions about their effectiveness^16^. Emerging studies, including randomized trials evaluating text-message support for young adult vapers, provide early evidence that vaping-tailored digital interventions can be beneficial^17^, yet rigorous evaluations of mobile apps designed specifically for e-cigarette cessation remain limited, especially in Middle Eastern populations. Given these gaps, the study aimed to evaluate changes in nicotine dependence among young adult exclusive e-cigarette users in Saudi Arabia following 30 days of engagement with a mobile cessation application.

## Methods

### Study design and setting

This exploratory, prospective, single-arm, pre-post study was conducted between December 2023 and March 2024 in Jeddah, Saudi Arabia, to evaluate the potential impact of a mobile application on e-cigarette cessation among young adults. The study employed a pre–post design in which each participant served as their own control. Ethical approval was obtained from the Institutional Review Board at King Abdullah International Medical Research Center (KAIMRC; IRB/2266/23), and all participants provided informed consent before enrollment.

### Participants

Eligible participants were young adults aged 18–40 years, current users of electronic cigarettes, residents of Saudi Arabia, and owners of smartphones capable of installing the intervention app, and able to read English. Individuals who used combustible cigarettes or dual tobacco products were excluded to ensure that changes in dependence could be attributed specifically to e-cigarette behavior. A total of 272 individuals completed the initial screening questionnaire. Of these, 123 met all eligibility criteria and were enrolled. Participants who completed both the pre-intervention and post-intervention assessments formed the final analytic sample (n=88). Only matched pre–post cases were used for inferential analyses (Figure 1).

**Figure 1:**
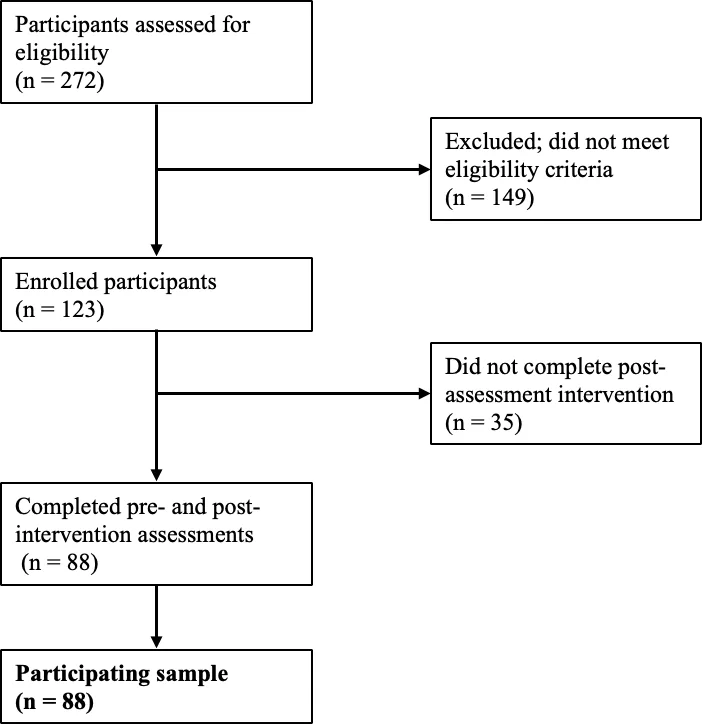
Flowchart of participant selection

### Sample size

Because the study involved a single-group pre–post design without a comparison arm, a formal hypothesis-driven power calculation was not applicable. Instead, enrollment targets were established to ensure adequate precision around estimates of within-person change in dependence scores. A minimum analytic sample of approximately 90 participants was planned, which provides sufficient precision to detect clinically meaningful within-person changes. For example, with 90 paired observations, a paired analysis has >90% power to detect a moderate standardized mean difference (Cohen’s d ≈ 0.4–0.5) in dependence scores at a two-sided α=0.05, consistent with effect sizes reported in previous digital cessation interventions. To account for expected attrition, a total of 123 participants were enrolled. Of these, 88 participants completed both the pre-intervention and post-intervention assessments, forming the final analytic sample.

### Sampling strategy

Participants were recruited using a non-probability convenience sampling strategy. An online demographic and screening questionnaire was disseminated via social media and institutional channels to reach young adult e-cigarette users. Individuals who completed the screening survey and met the preliminary criteria were contacted by phone and invited to participate. This approach approximates what is described as ‘probability convenience sampling’, but methodologically it is best classified as convenience sampling of eligible volunteers.

### Measures and scoring

The primary measure of dependence was the 10-item Penn State Electronic Cigarette Dependence Index (PS-ECDI)^18,19^, a validated scale assessing multiple dimensions of e-cigarette addiction: frequency of daily vaping, number of days used, urges and cravings, irritability and withdrawal symptoms, difficulty refraining in restricted places, nighttime awakening to vape, and time to first use after waking.

### Data collection procedures

Data were collected in two stages. Interested individuals completed an online screening and demographic questionnaire, which included age, gender, residence, ability to read English, smartphone ownership, and current e-cigarette use. Those meeting basic eligibility criteria were contacted by telephone. During this call, trained research staff obtained verbal confirmation of consent, administered the pre-intervention questionnaire (including PS-ECDI, GYTS^20,21^, and ITC items^22,23^, and assisted participants in downloading and setting up the Stay Quit Coach (SQC) mobile application. Participants were then instructed to enable notifications and to engage with the app over the ensuing month.

Follow-up and post-intervention assessment was performed approximately 1 month after baseline, participants were contacted again by phone or text message to schedule a follow-up call. During the follow-up call, the post-intervention questionnaire was administered, repeating the PS-ECDI. Participants who did not complete the follow-up questionnaire were classified as lost to follow-up (n=92) and were not included in the paired analyses.

### Scoring procedures

All scoring procedures were implemented in accordance with established methods from prior PS-ECDI validation studies and subsequent applications in the literature, ensuring methodological consistency with existing research^18-23^. Each item was scored according to the original validated ordinal scale (range 0–5 depending on item structure). Dichotomous responses (e.g. ‘Do you wake up at night to vape?’) were coded as 0=no and 1=yes. Items with reverse directionality were harmonized so that higher values consistently indicated higher dependence. The total PS-ECDI score was calculated as the sum of all 10 items, generating a continuous scale reflecting overall dependence.

Dependence categories followed established thresholds: not dependent (0–3), low dependence (4–8), medium dependence (9–12), high dependence (≥13). The PS-ECDI was administered identically before and after the intervention. No modifications to item wording or scoring rules were made.

### Global Youth Tobacco Survey (GYTS) items

Two items adapted from the WHO/CDC GYTS were included to characterize patterns of e-cigarette use. Days used in the past 30 days (scored on a 0–6 ordinal scale, capturing frequency of recent use) and recent attempts to quit (past 12 months) (scored 0=no, 1=yes, capturing cessation motivation). These items contextualized dependence severity and recent behavioral patterns.

### International Tobacco Control (ITC) survey item

One item adapted from the ITC Youth and Young Adult Survey assessed the recency of last use: responses were converted to a structured 4-level ordinal scale as follows (0 = ‘Within past week’; 1 = ‘Within past month’; 2 = ‘Within past 3 months’; and 3 = ‘More than 3 months ago’. This scale captured short-term behavioral changes relevant to cessation.

### Intervention: stay quit coach application

Participants were instructed to download and use the Stay Quit Coach app, an evidence-based mobile intervention developed by the US Department of Veterans Affairs. The app includes: daily logging of vaping behavior, motivational messages, craving management tools, breathing exercises, weekly consumption and cost tracking, and personalized cessation plans. Participants received a video tutorial on installation and use, followed by reminders sent via WhatsApp to promote engagement throughout the 30 day trial. A key feature of the Stay Quit Coach app is its integrated activity-tracking system, which automatically records the number of days a participant logs in, the timing of each log-in, and the extent of engagement with in-app modules. These backend usage metrics allowed researchers to objectively quantify adherence and assess patterns of interaction throughout the 30 day intervention period. These included the number of days a participant logged into the app, completion of behavioral modules, and interaction with tracking tools. For the analytic sample, login days ranged from 0 to 31, with a median of 20 days (IQR: 7–28). Approximately one-third of participants logged in on ≥27 days, whereas a smaller subset demonstrated minimal use (e.g. 0–5 days). Because item-level dependence outcomes were evaluated on a pre–post basis rather than through a dose–response framework, engagement metrics were summarized descriptively but not incorporated into inferential models. This exposure quantification provides context for interpreting intervention effects by indicating substantial variability in actual app engagement across users. To support sustained engagement, participants also received periodic WhatsApp reminders encouraging daily log-ins and completion of the app’s behavioral exercises. The app’s validated structure, ease of adaptation, and ability to track engagement made it a practical and theoretically grounded tool for evaluating digital cessation among young adults in Saudi Arabia, where culturally tailored vaping-specific apps remain scarce.

Although the Stay Quit Coach (SQC) application was originally developed for smoking cessation, it was selected for this study because its core behavioral components, craving management, stimulus control, coping planning, motivational reinforcement, and progress tracking target mechanisms that are shared across nicotine-delivery systems, including electronic cigarettes. Prior research shows that dependence on e-cigarettes and combustible tobacco is driven by overlapping constructs such as withdrawal, urges, habit strength, and reinforcement patterns, all of which are addressed within SQC’s behavioral framework. No culturally adapted vaping-specific mobile applications were available in Saudi Arabia at the time of study initiation; therefore, SQC represented the most evidence-based, structured, and scalable digital tool suitable for immediate implementation. Importantly, the app required no modification to administer the PS-ECDI or to support behavior change tasks relevant to e-cigarette users, and participants received standardized guidance on applying SQC strategies to vaping cues rather than smoking triggers.

### Data handling, preparation, and statistical analysis

Pre-intervention, post-intervention, and demographic datasets were merged using the participant’s unique study identifier. To ensure accuracy, all variables were checked for range validity and missingness. Categorical variables were recoded consistently across time points. No imputation was used; missingness was <5% for all variables. Only participants with complete paired pre–post responses across all dependence indicators, were included in repeated-measures analyses. All statistical analyses were conducted using R software (version 4.3.2; R Foundation for Statistical Computing). Demographic characteristics were summarized using frequencies and percentages. Prior to hypothesis testing, distributional assumptions for continuous dependence scores were evaluated using the Shapiro–Wilk test, visual inspection of Q–Q plots, and assessment of skewness and kurtosis. Although individual item scores exhibited non-normal distributions, the total dependence score demonstrated approximate normality, supporting the use of parametric repeated-measures methods.

For item-level analyses, the Wilcoxon signed-rank test was used to describe within-person changes between pre- and post-intervention responses. Effect sizes for all paired comparisons were estimated using Cohen’s d for paired samples, calculated as the mean of the within-person differences divided by their standard deviation (SD).

The primary evaluation of intervention impact was conducted using a repeated-measures analysis of variance (ANOVA) with a single within-participant factor (time: pre- vs post-intervention). This model provides an F statistic that is algebraically equivalent to the paired t-test and quantifies the overall main effect of time. Model assumptions, including normality of residuals, sphericity, and equality of variances, were assessed through residual diagnostics and variance-covariance inspection; violations of sphericity were not applicable because only two time points were compared.

To complement the ANOVA, a paired t-test was performed to estimate the mean difference (post–pre) with corresponding 95% confidence intervals (CIs). To explore whether baseline dependence predicted post-intervention dependence while accounting for regression to the mean, we additionally fit a baseline-adjusted linear regression model (ANCOVA framework) with post-intervention dependence as the outcome and baseline dependence as the sole predictor. Model assumptions of linearity, homoscedasticity, and normality of residuals were verified with standardized residual plots and Q–Q diagnostics. Results were reported as β coefficients with standard errors, 95% CIs, and two-sided p-values.

## Results

### Participant characteristics

A total of 88 participants completed the baseline assessment and completed the study period. Most were male (58; 65.9%) and aged 18–25 years (58; 65.9%). Participants resided predominantly in the Western region of Saudi Arabia (71; 80.6%), with smaller proportions from the Eastern (6; 6.8%), Riyadh (4; 4.5%), Southern (5; 5.6 %), and North (2; 2.2 %) regions. Nearly all participants had not enrolled in a vaping cessation program in the preceding 30 days (85; 96.5%), although most reported at least one quit attempt in the past year (55; 62.5%). Recent e-cigarette use was common: 61 participants (69.3%) reported using an e-cigarette within the day, and 23 (26.1%) within the past week. Only 3 participants (3.4%) used an e-cigarette within the past 30 days, and 1 participant (1.14%) did not use it for more than 30 days. Most participants reported frequent or near-daily use, with 62 individuals (70.4%) vaping on 20 to 30 days in the previous month and 15 (17.05%) vaping on 10 to 19 days. Daily consumption intensity was also high; 18 participants (20.4%) reported using an e-cigarette more than 30 times per day, while 16 (18.1%) used it 20 to 29 times per day. At baseline, 41 participants (26.5%) of the sample met criteria for high dependence on the PS-ECDI, and more than half demonstrated medium or low dependence (49; 55.6%). Overall, the sample comprised young, frequent vapers with substantial nicotine dependence and little recent exposure to formal cessation resources, highlighting the need for accessible mobile interventions in this population (Table 1).

**Table 1:** Baseline demographic and vaping-related characteristic of the participants (N=88)

Characteristics	n (%)
**Gender**	
Male	58 (65.9)
Female	30 (34.1)
**Age** (years)	
18–25	58 (65.9)
26–40	30 (34.1)
**Region**	
Western	71 (80.6)
Eastern	6 (6.8)
Riyadh	4 (4.5)
Southern	5 (5.6)
North	2 (2.2)
**Registered in a cessation program** (past 30 days)	
No	85 (96.5)
Yes	3 (3.4)
**Attempted to quit in the past 12 months**	
Yes	55 (62.5)
No	33 (37.5)
**Last e-cigarette use**	
Within the day	61 (69.3)
Within the past 7 days	23 (26.1)
Within the past 30 days	3 (3.4)
More than 30 days	1 (1.14)
**Days used e-cigarettes in the past 30 days**	
1–2	4 (4.5)
3–9	7 (7.9)
10–19	15 (17.1)
20–30 (daily/almost daily)	62 (70.5)
**Times per day use electronic cigarette**	
0–4	14 (15.9)
5–9	20 (22.7)
10–14	13 (14.7)
15–19	7 (7.9)
20–29	16 (18.1)
≥30	18 (20.4)
**Baseline EC dependence (PS-ECDI)**	
High	26 (29.5)
Medium	26 (29.5)
Low	23 (26.1)
Not dependent	13 (14.7)

*One ‘time’ consists of around 15 puffs or lasts around 10 minutes.

### E-cigarette brand distribution

Among participants who reported their primary e-cigarette brand, Mazaj was the most commonly used device (54; 28.0%), followed by brands classified as Other (39; 20.2%). IDK and Caliburn KOKO were each used by 29 participants (15.0%). Less frequently reported brands included Wenax (21; 10.9%), Oxva (14; 7.3%), and Elfbar (7; 3.6%).

### Changes in vaping-dependence indicators

Among 88 participants with complete paired data, substantial reductions were observed across multiple vaping-dependence behaviors over the 1 month intervention period. The number of days participants used e-cigarettes in the past 30 days was significantly reduced by 16.7% (p<0.01), and daily use episodes decreased by 41.4% (p<0.01). Participants reported significant reductions in cravings, urge strength, irritability, restlessness, and difficulty refraining from vaping in restricted areas (all p<0.001). Nighttime awakening to vape was significantly decreased by 62.5% (p=0.02). The only indicator that increased was the time to first vape after waking, which significantly rose by 47.3% (p<0.01), reflecting a delay in morning nicotine intake (Table 2, Figures 2 and 3).

**Table 2:** Changes in vaping-dependence indicators from pre- to post-intervention among participants who completed both assessments

Question	N pairs	Pre mean (SD)	Post mean (SD)	Mean diff (Post-Pre)	% change vs Pre	Cohen d (paired)	p (paired t-test)
During past 30 days, on how many days did you use electronic cigarettes/vape?	88	3.53 (0.83)	2.94 (1.10)	−0.59	−16.7	0.64	<0.00001
On days that you can use your electronic cigarette freely, how soon after you wake up do you first use your electronic cigarette?	88	2.98 (1.86)	4.39 (1.78)	1.41	47.3	0.78	<0.00001
Do you sometimes awaken at night to use your electronic cigarette?	88	0.09 (0.29)	0.03 (0.18)	−0.06	−62.5	0.24	0.0245
If yes, how many nights per week do you typically awaken to use your electronic cigarette?	88	1.12 (0.45)	1.05 (0.30)	−0.08	−7.1	0.23	0.0340
Do you use an electronic cigarette now because it is really hard to quit it?	88	0.52 (0.50)	0.36 (0.48)	−0.16	−30.4	0.30	0.0054
Do you ever have strong cravings to use an electronic cigarette?	88	0.77 (0.42)	0.57 (0.50)	−0.20	−26.5	0.40	0.0003
Over the past week, how strong have the urges to use an electronic cigarette been?	88	2.12 (0.71)	1.72 (0.64)	−0.41	−19.3	0.50	<0.00001
Is it hard to keep from using an electronic cigarette in places where you are not supposed to?	88	0.44 (0.50)	0.20 (0.41)	−0.24	−53.8	0.50	<0.00001
Did you feel more irritable because you couldn’t use an electronic cigarette?	88	0.43 (0.50)	0.22 (0.41)	−0.22	−50.0	0.42	0.0002
Did you feel nervous, restless, or anxious because you couldn’t use an electronic cigarette?	88	0.41 (0.49)	0.25 (0.44)	−0.16	−38.9	0.28	0.0098

Item-level means appear low because several PS-ECDI components are dichotomous (0–1) or use restricted ordinal ranges, and behaviors such as nighttime awakening are uncommon in this population. These values represent question-by-question scoring rather than the full summed dependence scale. p<0.05 is considered statistically significant.

**Figure 2:**
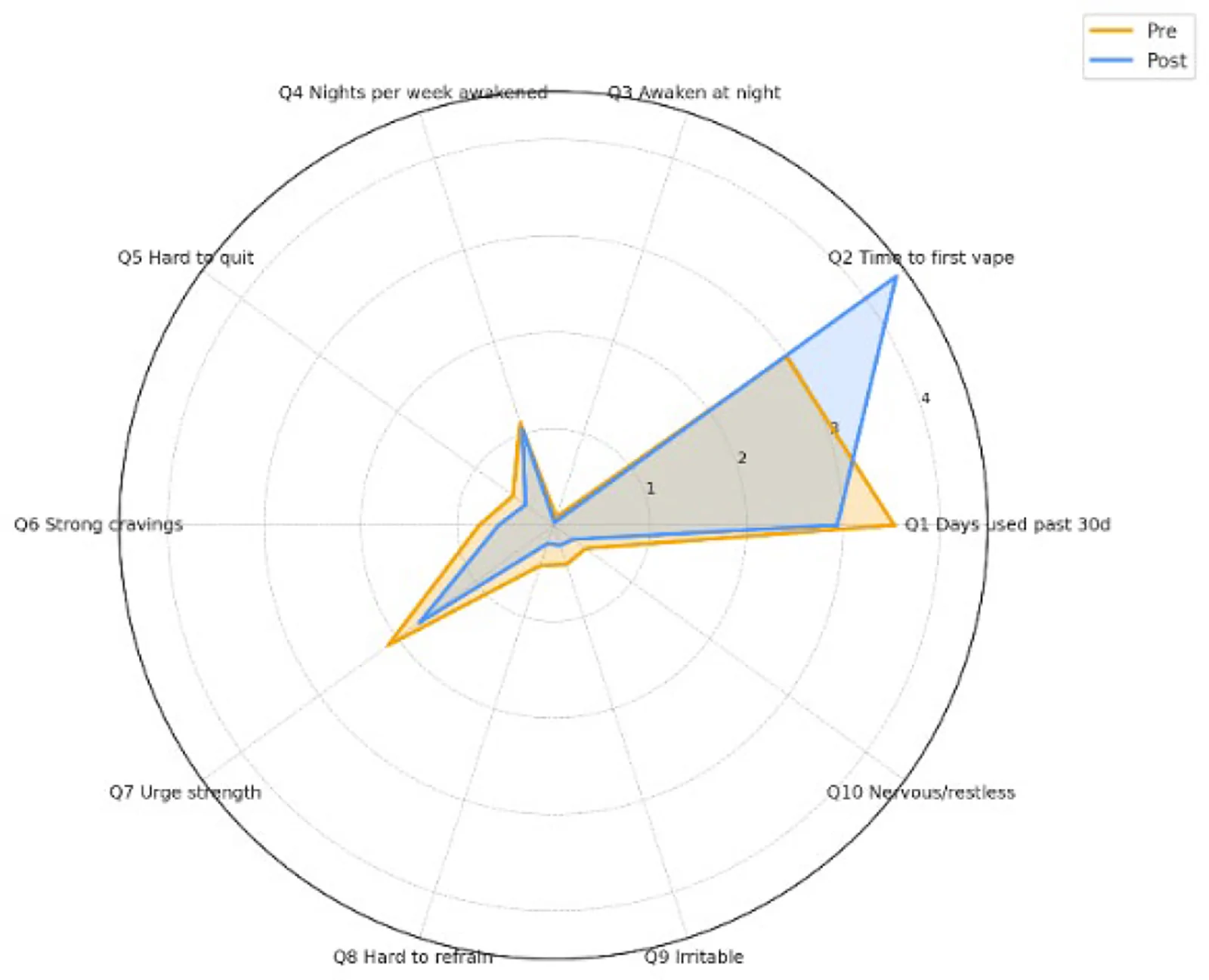
Pre- vs post-intervention dependence scores across all indicators

Higher values reflect greater vaping dependence. Each axis represents a single ordinal dependence item scored according to the study’s standardized coding schema (range: 0–4 per item). Shaded areas illustrate overall reduction in dependence from baseline to follow-up.

**Figure 3:**
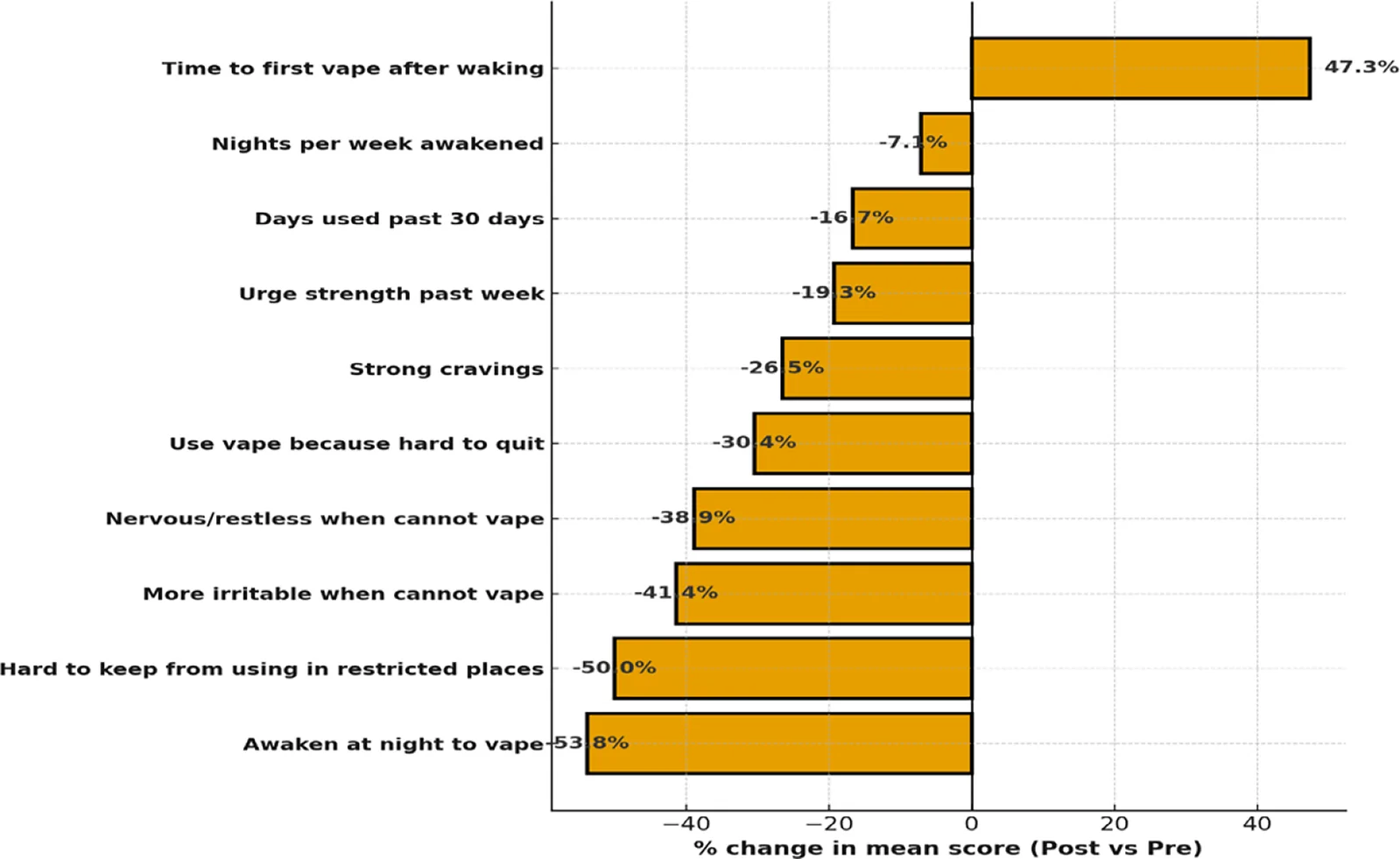
Percentage change in vaping-dependence indicators from pre- to post-intervention

**Footnote:** The percentage change was calculated as [(Post − Pre) / Pre] × 100% for each dependence indicator. Negative values indicate reductions in vaping dependence following the intervention, whereas positive values indicate increases. The indicators are arranged in descending order of improvement.

The total PS-ECDI score decreased from a mean of 9.5 to 5.4 following the intervention, representing a 43.2% reduction. The mean difference of -4.1 points was statistically significant (95% CI: -5.0 – -3.3). Both paired t-tests and Wilcoxon tests confirmed the robustness of this decline. This consistent improvement across parametric and nonparametric analyses suggests that the intervention produced a clinically meaningful reduction in overall nicotine dependence, not merely an isolated or item-specific effect.

### Repeated measure analysis

Among the 88 participants with complete paired dependence assessments, vaping-dependence scores decreased substantially following the intervention. Mean dependence declined from 9.48 (SD=5.12) at baseline to 5.40 (SD=3.81) post-intervention, corresponding to a mean within-person reduction of -4.08 points (95% CI: -5.04 – -3.12), representing a 43.0% relative decrease. A paired t-test demonstrated that this reduction was statistically significant (t₈₇ = -9.46; p<0.001). The repeated-measures ANOVA confirmed a significant main effect of time [F(1, 87) = 89.45; p<0.001]. The magnitude of improvement was large, as reflected by the effect size (Cohen d=1.01) (Table 3; and Supplementary file Figures 1 and 2).

**Table 3:** Baseline-adjusted linear regression (ANCOVA) predicting post-intervention dependence scores from baseline dependence

Term	β	SE	t	p	95% CI
Intercept	0.998	0.673	1.48	0.142	−0.34–2.34
Pre score	0.464	0.063	7.42	<0.001	0.34–0.59

R^2^=0.39 (39% of variance in post scores explained by pre). F(1, 86)=55.06, p=7.76×10^-11^. Statistically significant at p<0.05.

## Discussion

In this prospective, pre-post assessment of young adult e-cigarette users in Saudi Arabia, 1 month of engagement with a mobile cessation application was associated with substantial reductions across multiple dimensions of vaping dependence. The sample population reported predominantly using high-nicotine pod-based systems, which are typically associated with stronger nicotine delivery and higher dependence.

Participants reported fewer days of vaping, fewer daily use episodes, and marked declines in cravings, withdrawal symptoms, and difficulty refraining in restricted settings. These findings demonstrate meaningful behavioral improvement across several dimensions of dependence, consistent with an effective reduction in nicotine-driven use. Furthermore, the 43% reduction in the PS-ECDI total score represents a clinically meaningful improvement in dependence severity and contributes new regional evidence to a global literature in which data on vaping-specific digital cessation tools remain limited. The magnitude of improvement aligns with findings from earlier work using the Penn State Electronic Cigarette Dependence Index. Foulds et al.^18^, who developed and validated the PS-ECDI, demonstrated that higher scores are strongly correlated with greater frequency and intensity of use, two behavioral parameters that declined substantially in our study population. Similarly, previous studies have reported moderate to high reductions in dependence scores following cessation attempts among motivated users, although these samples primarily comprised Western populations^24^. Our findings extend this literature by demonstrating comparable or greater improvements within a Middle Eastern population using a fully digital, remotely administered intervention. Reductions in craving intensity, irritability, restlessness, and nighttime awakening parallel patterns observed in studies evaluating behavioral markers of nicotine dependence^20-23^.

However, our results differ from several meta-analyses of digital cessation tools, which have generally reported mixed or modest effects and, in some cases, insufficient evidence of effectiveness^25-27^. A recent youth-focused meta-analysis found no significant abstinence benefit for app-based interventions compared with minimal support, although text-message programs showed more reliable outcomes^28^. These discrepancies underscore the heterogeneity of digital interventions; effectiveness likely varies according to app design, behavioral content, interactivity, and user engagement. The relatively pronounced reductions in our sample may reflect the structured behavioral components within the Stay Quit Coach app, including craving-management tools, reinforcement messaging, and goal-monitoring features not consistently included in commercially available applications evaluated in prior reviews.

Contextual factors in Saudi Arabia may also have influenced outcomes. Unlike many Western samples in which dual use of cigarettes and e-cigarettes is common, our eligibility criteria restricted enrollment to exclusive e-cigarette users, enabling more targeted behavior change. Participants were predominantly young, highly motivated to quit, and had limited prior exposure to formal cessation resources, conditions that may enhance responsiveness to behavioral interventions. High baseline motivation may partially explain the larger magnitude of change compared with meta-analytic averages drawn from more heterogeneous populations.

Several factors temper the interpretation of these findings. The absence of a control group limits causal inference, and observed improvements may partly reflect self-monitoring, regression to the mean, or unmeasured confounding. These limitations mirror concerns raised in prior digital-intervention reviews. Nevertheless, the consistency and magnitude of reductions across nearly all dependence indicators strengthen the plausibility of a genuine intervention effect. The concordance of results from parametric and nonparametric analyses, along with dose-response behavioral markers such as delayed morning use and fewer nighttime awakenings, further support meaningful behavior change. Additionally, the short 30 day intervention period restricts the ability to evaluate long-term abstinence or relapse trajectories.

This study helps to address a critical evidence gap by providing one of the first evaluations of vaping-cessation outcomes among Arabic-speaking young adults. Digital cessation research in Saudi Arabia is limited, and no prior studies have reported pre- to post-intervention PS-ECDI changes in this population. Given the high rates of smartphone ownership and rising e-cigarette use among Saudi youth, mobile-health approaches offer a scalable means to expand cessation support where culturally tailored programs remain scarce.

### Limitations

The study has several limitations. All vaping measures were self-reported, which may introduce social desirability bias and recall bias, biochemical verification was not feasible, and attrition may introduce response bias. In addition, the relatively small sample size (88 participants with paired pre–post responses), the focus on a specific age range (18–40 years), and the use of convenience sampling limit the generalizability of the findings. The geographical distribution of participants was also unbalanced, with most participants recruited from the Western region of Saudi Arabia, which may further limit the applicability of the results to the broader Saudi population. Moreover, the app was originally developed for smoking cessation and may not fully address e-cigarette-specific behavioral triggers. The application was not specifically culturally adapted for the Saudi population, which may have influenced user engagement and the overall effectiveness of the intervention. Future research should incorporate randomized controlled designs, culturally tailored or locally adapted content, integration with pharmacotherapy, and extended follow-up to assess the durability of effects. Despite these limitations, the findings suggest that a structured mobile application can meaningfully reduce vaping dependence among young adults in Saudi Arabia.

## Conclusions

In this prospective assessment of young adult e-cigarette users in Saudi Arabia, a 1 month mobile health intervention was associated with substantial reductions in multiple markers of vaping dependence, including frequency of use, craving intensity, withdrawal symptoms, and difficulty refraining in restricted settings. The overall PS-ECDI score decreased by more than 40%, indicating a clinically meaningful decline in nicotine dependence. Improvements were observed across nearly all behavioral indicators and were consistent in both parametric and nonparametric analyses. Although individuals with higher baseline dependence and male participants exhibited higher residual scores, the intervention demonstrated effectiveness across demographic subgroups. These findings provide early but important evidence that mobile applications may serve as accessible and scalable tools for vaping cessation in settings where culturally adapted services remain limited. As e-cigarette use continues to rise among young adults in the region, digital health interventions offer a practical approach to expanding cessation support. Larger randomized trials, extended follow-up, and further tailoring of content to the behavioral patterns of e-cigarette users are warranted to confirm the durability of effects and to optimize app-based cessation strategies.
