# Supplementary material for: Short-term reduction in e-cigarette dependence using a mobile health app
Source: Tob Prev Cessat. 2026 Oct 15;12:10.18332/tpc/225571. doi: 10.18332/tpc/225571 (PMC13715561; doi:10.18332/tpc/225571)
Supplement: Supplementary file 1 [file TPC-12-64-s001.pdf]

TOBACCO PREVENTION AND CESSATION

Supplementary file

© 2026 Alodaini S. et al.

DOI:
10.18332/tpc/225571

The content has been provided by the author(s) and has not been reviewed, verified, or endorsed by European Publishing. It may not have undergone peer review. The views, opinions, and recommendations expressed are solely those of the author(s) and do not necessarily reflect the position of European Publishing. European Publishing accepts no responsibility or liability for any consequences arising from the use of, or reliance on, this content.

SUPPLEMENTARY FILE

Supplementary Figure 1. Distribution of pre- and post-intervention dependence scores

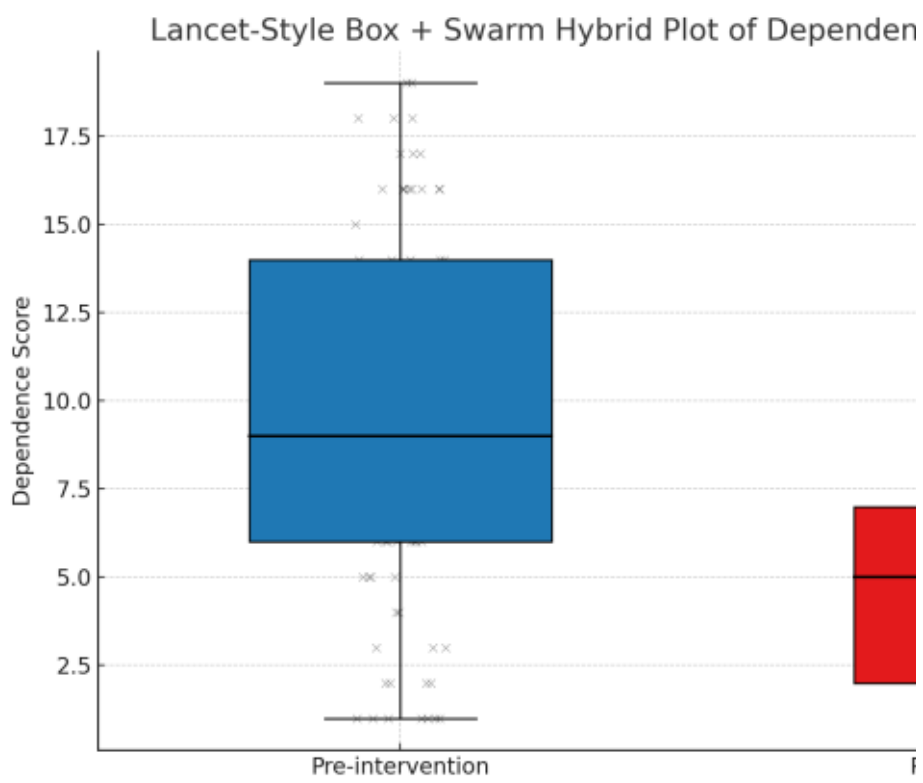

Supplementary Figure 2. Individual pre–post trajectories of vaping-dependence scores

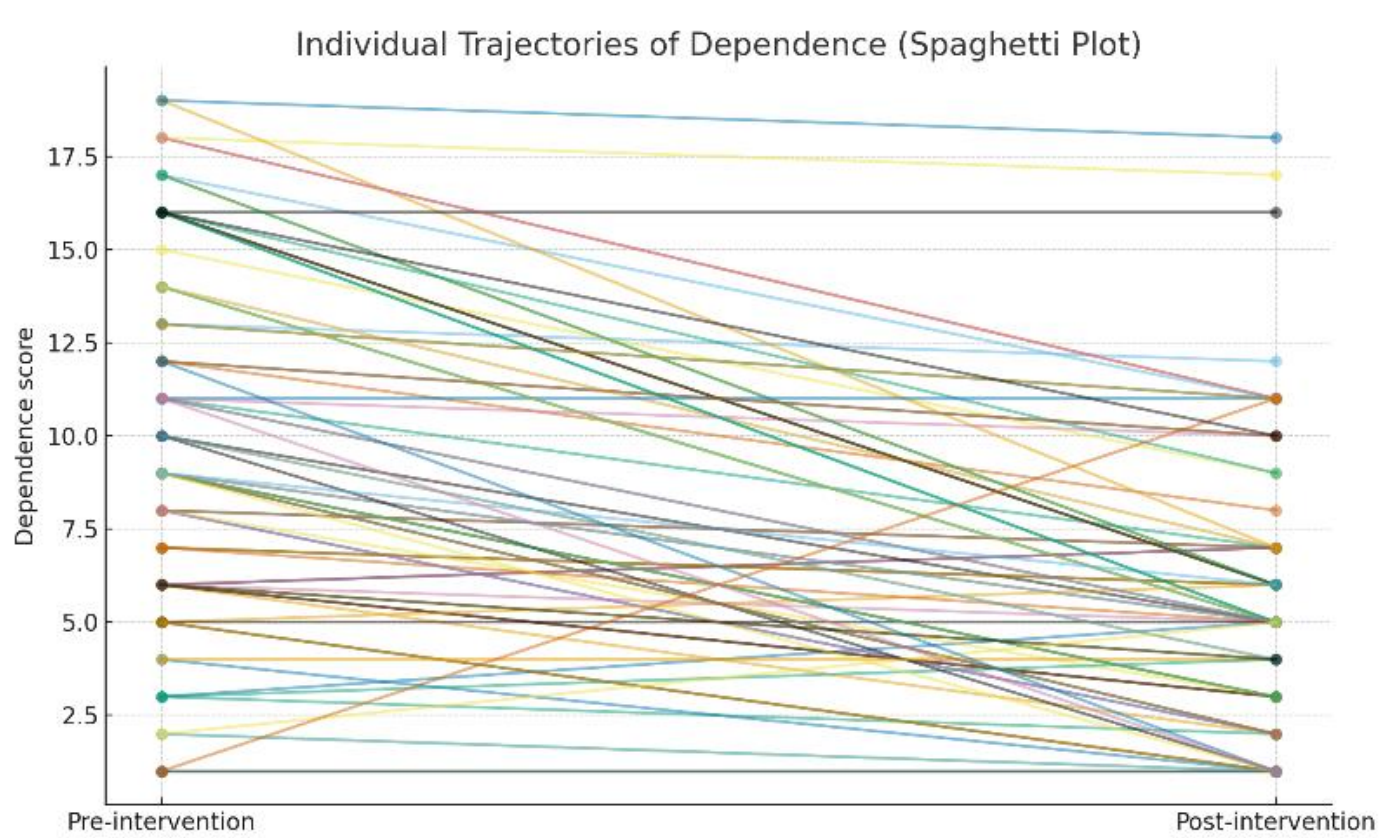

Each line represents a single participant's dependence score measured at baseline and follow-up. Downward-sloping lines indicate reductions in vaping dependence following the intervention. Scores reflect the standardized total dependence scale used in the study
